# Lifestyle in adulthood can modify the causal relationship between BMI and islet function: using Mendelian randomization analysis

**DOI:** 10.1186/s13098-022-00828-7

**Published:** 2022-04-21

**Authors:** Xuekui Liu, Huihui Xu, Ying Liu, Manqing Yang, Wei Xu, Houfa Geng, Jun Liang

**Affiliations:** 1 Department of Central Laboratory, Xuzhou Central Hospital, Xuzhou, Jiangsu China; 2 Department of Operating room, Xuzhou City Hospital of TCM, Xuzhou, Jiangsu China; 3 Department of Ultrasonography, Xuzhou Central Hospital, Xuzhou, Jiangsu China; 4 Department of Endocrinology, Xuzhou Central Hospital, Xuzhou, Jiangsu China

**Keywords:** Mendelian randomization, BMI, Islet function, Insulin resistance

## Abstract

**Background:**

Body mass index was intimately associated with islet function, which was affected by various confounding factors. Among all methods of statistical analysis, Mendelian randomization best ruled out bias to find the causal relationship. In the present study, we explored the relationship between 13 East Asian body mass index-related genes reported previously and islet function using the Mendelian randomization method.

**Methods:**

A total of 2892 participants residing in northern China were enrolled. Anthropological information, such as sex, age, drinking status, smoking status, weight, height and blood pressure, was recorded for all participants. Fasting glucose and insulin were detected, and the insulin sensitivity index was calculated. 13 single nucleotide polymorphismss in East Asian body mass index -related genes were analysed with the ABI7900HT system.

**Results:**

Five genetic locus mutations, CDKAL1, MAP2K5, BDNF, FTO and SEC16B, were found to be associated with body mass index and were used to estimate the genetic risk score. We found that the genetic risk score was negatively associated with the insulin sensitivity index. Even after adjusted of confounding factors, the relationship showed statistical significance. A subsequent interaction effect analysis suggested that the negative relationship between the genetic risk score and insulin sensitivity index no longer existed in the nondrinking population, and smokers had a stronger negative relationship than nonsmokers.

**Conclusion:**

We found a negative causal relationship between body mass index-related genetic locus mutations and insulin resistance, which might be increased by acquired lifestyle factors, such as drinking and smoking status.

## Introduction

Insulin resistance (IR), a metabolic disorder, has been described as an epidemic in the obese population. IR is an important pathomechanism of type 2 diabetes mellitus (T2DM) [1], and it is also a risk factor for cancer [2], Alzheimer’s disease [3], polycystic ovarian syndrome [4], and cardiovascular disease [5]. Eighty percent of obese individuals have IR symptoms, and obesity-induced chronic proinflammation contributes to the development of IR. Previous studies [6, 7] have shown that obesity plays a pivotal role in the development of IR. However, there are some limitations in traditional studies exploring the association between obesity and IR.

Classical research methods, such as cross-sectional studies and case–control studies, have lower credibility in determining causal relationships [8]. These study methods cannot fully control the influence of confounding factors, which may be unknown and cannot be measured [9]. A cohort study is a good method to verify the association between obesity and IR, but this type of study consumes too much time, capital and energy. Experimental research approaches are only carried out in the laboratory and use animals as samples, and the correlation between obesity and IR in humans cannot be observed. Therefore, a new research approach in humans that reflects a causal relationship will provide a greater of awareness of IR for medical care. In addition, the interaction effect is an important method to explore the association between three variables in observational studies. However, disturbance, from an unknown factor or confounding factor, makes the interaction lose its authenticity.

Mendelian randomization (MR) is a new approach to genetic analysis that utilizes genetic loci as instrumental variables to explore the relationship between exposure factors and outcomes [10]. This method makes use of the randomization process of Mendelian laws of genetics to exclude disturbances from unknown factors and confounding factors in observational studies and can better exhibit the causal relationship between exposure factors and outcomes [11]. Simultaneously, MR provides us with a new approach to analyse the interaction effect, i.e., the effect of the exposure factor and covariate on the outcome.

In this study, we examined 13 genetic loci reported by Wen W that were associated with body mass index (BMI) in East Asians [12]. We wanted to verify the causal relationship between BMI and islet function and to explore the effect of the interaction between BMI and other covariates on islet function.

## Subjects and methods

### Study populations

The study included a total of 2892 participants who lived in the city of Xuzhou, North China, from December 2020 to February 2021. This population in this community has regular physical examinations in Xuzhou Central Hospital. Individuals who met the inclusion criteria and exclusion criteria were recruited for this study. The inclusion criteria were as follows: (1) individuals were ≥ 18 years old and (2) individuals volunteered to participate in the study. The exclusion criteria were as follows: (1) type 1 diabetes mellitus; (2) acute complications from diabetes, such as diabetic ketoacidosis, a hypoglycaemic hypersomnia state, lactic acidosis or hypoglycaemic coma; (3) pregnancy; or (4) heart failure, haematologic disease, liver dysfunction, or steroid use. Because our sample was from the community, some T2DM patients with newly diagnosed were selected into this study inevitably. For controlling the heterogeneity of the participants, on the basis of T2DM diagnostic criteria of the American Diabetes Association [13], the T2DM patients, who were diagnosed less than 3 months, would be included into this study finally. In total, 2268 individuals were included in the present study.

### General examinations

All participants were surveyed to collect information on sex, age, drinking status, smoking status, weight, height and blood pressure before the next examination. These covariates were measured by trained medical workers. Sex, age, and whether the subjects had a history of drinking or smoking were acquired by a questionnaire. Participants who “drank alcohol more than five times in 1 year and consumed more than 20 ml of alcohol every time in the past year” were defined as “drinking,” while others were defined as “non-drinking.” Participants who “smoked more than one cigarette per day and maintained this status over 1 year” were defined as “smokers,” and others were defined as “non-smokers.” Weight was recorded to the nearest 0.1 kg when the participants wore light clothing and no coat or shoes. After individuals rested for at least 5 min, systolic pressure and diastolic pressure were measured using a semiautomatic pressure monitor. Biomarker levels, including fasting glucose and fasting insulin, were measured by venous blood sampling. Blood was drawn from every participant after fasting overnight (8 ~ 10 h). BMI was calculated according to the following formula: BMI = weight (kg)/height^2^ (m). Insulin resistance was evaluated using homeostasis model assessment (HOMA), with the formula as follows: HOMA-IR = fasting glucose (mmol/l) × fasting insulin (mU/l)/22.5 [14]. Beta cell function was evaluated using HOMA-β, with the formula as follows: HOMA-β = [20 × fasting insulin (mU/l)/[fasting glucose (mmol/l)–3.5] [15]. The formula for the insulin sensitivity index was as follows: IAI = 1/[fasting glucose (mmol/l) × fasting insulin (mU/l)] [16].

### Genotyping

Thirteen genetic loci related to BMI in East Asians reported by Wang W (PCSK1, GP2, GLPR/QPCTL, PAX6, ADCY3/RBJ, MC4R, GNPDA2, CDKAL1, MAP2K5, BDNF, FTO, SEC16B and TFAP2B) were detected in this study. The detection of all SNPs was performed using TaqMan SNP allelic discrimination on an ABI7900HT system (Applied Biosystems, Foster City, CA). The detected SNP alleles were transformed to numbers according to previous genome-wide association studies. We also analysed the relationship between 13 genetic loci and BMI, and some relationships with BMI in our sample were calculated using the genetic risk score (GRS). This is the first step of MR. Furthermore, the association between the GRS and outcome variables was analysed to verify the relationship between BMI and outcome variables.

### Statistical analysis

Data management and analysis were performed using SAS statistical software (Version 9.3; SAS Institute, Inc., Cary, NC, USA). Descriptive statistics revealed the general clinical characteristics of the participants. Linear regression was performed to analyse the association between 13 genetic loci and BMI. Pearson’s correlation and logistic regression were used to examine the relationship between islet function and GRS. All reported P values are two-tailed. The level of statistical significance was set at *P* < 0.05.

## Results

### The clinical characteristics of all participants

In the present study, 2268 individuals were included in the final analysis (Table 1). The average age of the participants was 46.40 years, and the average BMI was 24.75 kg/m^2^. This study had 1105 males and 1163 females. Islet function, which included HOMA-β, HOMAIR and the IAI, was estimated by FPG and Fins. Our sample included 1516 individuals who drank alcohol and 752 nondrinking participants. Smokers made up 48.64% (1103/2268) of the study participants and non-smokers made up 51.46% (1165/2268) of the participants. 31(1.37%) T2DM patients with newly diagnosed less than 3 months were included into this study.

**Table 1:** The clinical characteristics of participants

Variables	Mean (SD) or frequency (%)
n	2268
Age (years)	46.40 (9.47)
Male	1105 (48.72%)
BMI (Kg/m^2^)	24.75 (3.08)
SBP (mmHg)	125.06 (15.92)
DBP(mmHg)	79.92 (11.46)
FPG(mmol/L)	5.28 (1.26)
Fins (u/dl)	9.72 (7.17)
HOMAβ	634.91 (407.34)
HOMAIR	2.36 (2.67)
IAI (× 10)	2.76 (1.84)
T2DM	31 (1.37%)
Smoking (%)	1103 (48.63%)
Drinking (%)	1516 (66.84%)

*BMI* body mass index, *SBP* systolic blood pressure, *DBP* diastolic blood pressure, *FPG* fasting plasma glucose, *Fins* fasting insulin, *HOMAβ* homeostasis model assessment β, *HOMAIR* homeostasis model assessment insulin resistance, *IAI* insulin sensitivity indexes, *T2DM* type 2 diabetes mellitus

### The association between SNPs and BMI

The first step of MR was to verify the association between the SNPs that we detected and BMI. Before analysis, the GRS was calculated according to the number of alleles associated with increased BMI. Table 2 provides this correlation for all participants. In our samples, five SNPs (from CDKAL1, MAP2K5, BDNF, FTO and SEC16B) were related to BMI, and the other genes (PCSK1, GP2, GLPR/QPCTL, PAX6, ADCY3/RBJ, MC4R, GNPDA2 and TFAP2B) were not associated with BMI. Using the five SNPs and their corresponding β values, we estimated the GRS for subsequent analysis. The formula for the GRS is as follows:

$$ GRS = \frac{{w_{1} SNP_{1j} + w_{2} SNP_{2j} + \cdots + w_{m} SNP_{mj} }}{{w_{1} + w_{2} + \cdots + w_{m} }} $$

**Table 2:** The association between SNPs and BMI in the participants

SNP	Nearest gene	Effect allele	Other allele	MAF	β	95% CI	P
rs9356744	CDKAL1	T	C	0.577	0.183	0.361 ~ 0.004	0.045
rs261967	PCSK1	C	A	0.402	0.122	0.059 ~ 0.302	0.187
rs12597579	GP2	C	T	0.721	0.136	0.331 ~ 0.058	0.169
rs11671664	GIPR/QPCTL	G	A	0.515	0.035	0.214 ~ 0.144	0.701
rs4776970	MAP2K5	A	T	0.245	0.427	0.227 ~ 0.627	< 0.001
rs6265	BDNF	C	T	0.535	0.276	0.103 ~ 0.450	0.002
rs652722	PAX6	T	C	0.358	0.003	0.183 ~ 0.188	0.978
rs6545814	ADCY3/RBJ	G	A	0.44	0.084	0.091 ~ 0.259	0.346
rs17817449	FTO	G	T	0.124	0.447	0.184 ~ 0.710	0.001
rs6567160	MC4R	C	T	0.213	0.118	0.098 ~ 0.333	0.284
rs574367	SEC16B	T	G	0.232	0.381	0.167 ~ 0.594	< 0.001
rs10938397	GNPDA2	G	A	0.298	0.161	0.032 ~ 0.352	0.102
rs4715210	TFAP2B	T	C	0.165	0.038	0.199 ~ 0.275	0.316

where ω is the β value corresponding to the SNP.

### GRS was associated with islet function

**Fig. 1:**
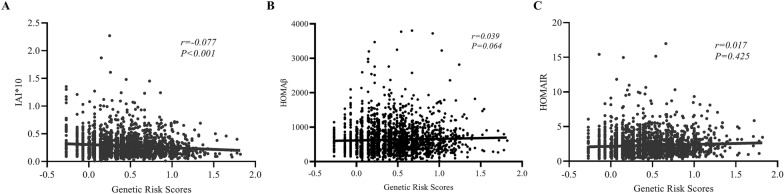
The correlation between genetic risk score and indices of islet function (**A** a scatter plot with the IAI(× 10) and GRS; **B** a scatter plot with HOMAβ and the GRS; **C**: a scatter plot with HOMAIR and the GRS)

As shown in Fig. 1, there was a negative correlation between the GRS and the IAI, an index of insulin sensitivity (*P* < 0.001). The relationship between the GRS and HOMAIR was not significant, and the GRS was also not associated with HOMAβ.

### Linear regression was conducted to explore the association between the GRS and islet function

Table 3 shows that there was no correlation between GRS and HOMA-IR or HOMA-β in this sample, but the IAI will always be associated with the GRS if BMI is not adjusted. The IAI result verified assumption 3 of MR, that genes act on the outcome only by influencing the exposure.

**Table 3:** The association between genotic risk scores and indexs of islet function

		Beta	95% CI	t	P
HOMAβ	Model1	44.471	2.522 ~ 91.463	1.856	0.064
	Model2	47.416	0.059 ~ 94.241	1.916	0.052
	Model3	41.969	4.698 ~ 88.635	1.764	0.078
	Model4	10.296	34.474 ~ 55.067	0.451	0.652
HOMAIR	Model1	0.126	0.183 ~ 0.435	0.798	0.425
	Model2	0.085	0.222 ~ 0.393	0.545	0.586
	Model3	0.035	0.269 ~ 0.339	0.223	0.823
	Model4	0.117	0.415 ~ 0.181	0.767	0.443
IAI(× 10)	Model1	0.04	0.061 ~ 0.018	3.633	< 0.001
	Model2	0.036	0.057 ~ 0.015	3.346	< 0.001
	Model3	0.03	0.051 ~ 0.01	2.877	0.004
	Model4	0.012	0.031 ~ 0.007	1.221	0.223

*Model1* unadjusted; *Model2* adjusted sex, age; *Model3* adjusted sex, age, SBP, DBP, drunking status; *Model4* adjusted sex, age, SBP, DBP, drunking status, smoking status and BMI

### The measurement data IAI*10 were transformed into two-category data according to the lower quarter value of IAI*10; the value of IAI*10 < 0.16 was set as 1, and the others were set as 0

**Fig. 2:**
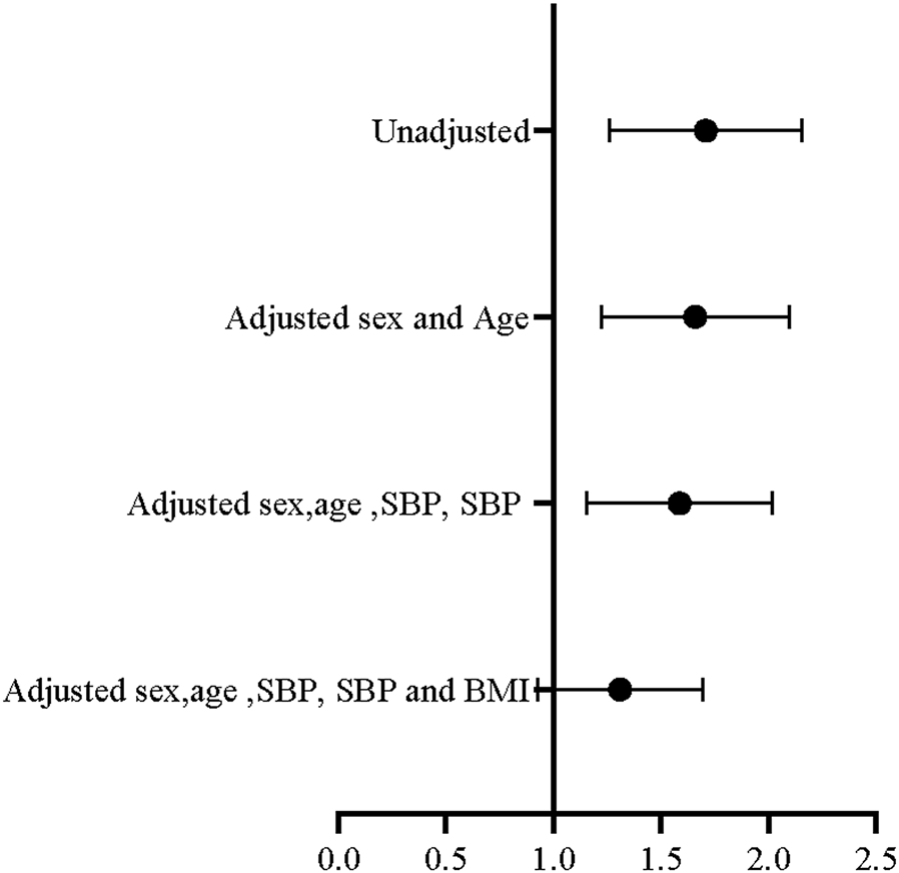
The association between genetic risk score and lower IAI*10 in the study participants

Figure 2 shows the OR value of the GRS for lower IAI*10 using logistic regression. When the logistic regression equation included the BMI variable, the GRS was not significant in the formula. This result conformed to the assumptions of MR (Fig. 2).

### The interaction effect analysis

**Fig. 3:**
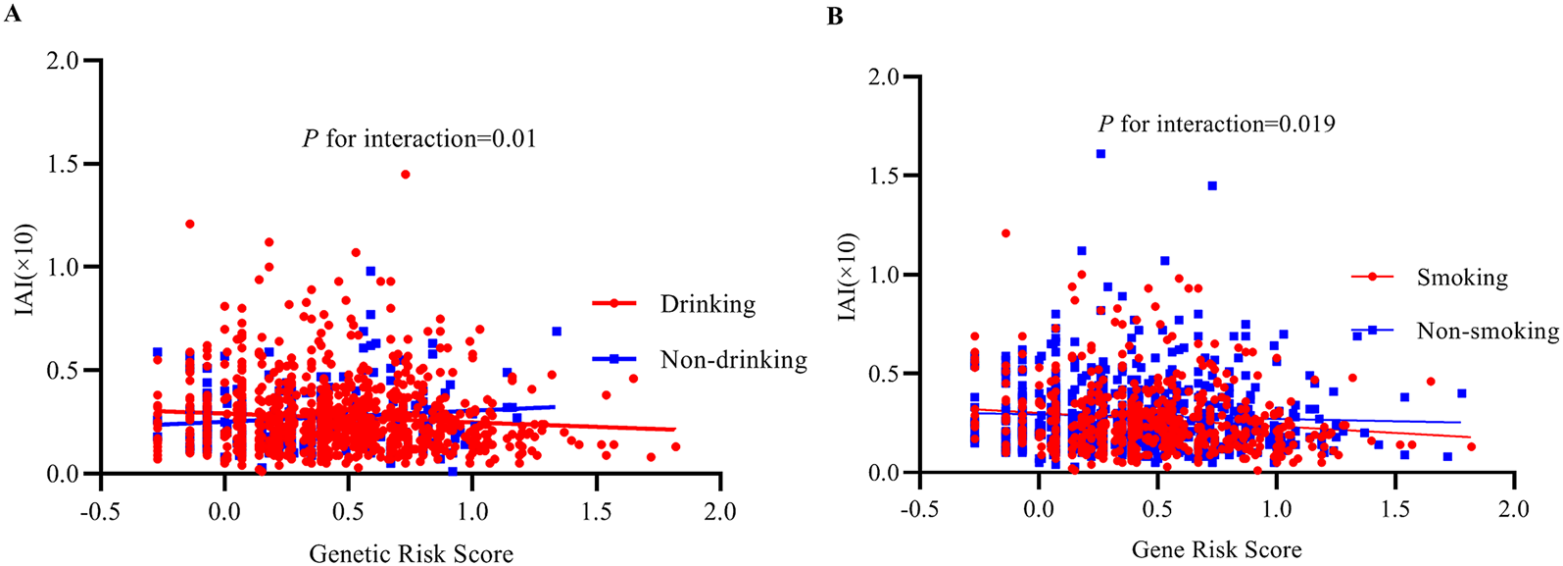
Drinking status and smoking status modified the association between the GRS and IAI

Drinking status and smoking status modified the association between the GRS and IAI. Figure 3A shows that the IAI*10 for drinking individuals decreased with increasing GRS, but this trend was not observed in the nondrinking participants. Interestingly, data in this figure demonstrate an interaction effect between drinking status and GRS on the IAI*10, with a *P* value of 0.01. Smoking status also modified this association. Figure 3B shows that there was a stronger negative relationship between GRS and IAI*10 in the smoking population, and this relationship was weak in non-smoking individuals (*P* = 0.019).

## Discussion

In the present study, we performed MR to verify the causal relationship between BMI and islet function and to explore the effect of the interaction between BMI and covariates (lifestyle factors that included drinking status and smoking status) on islet function. Our results show that BMI was a significant reason for decreased insulin sensitivity in Chinese adults, and lifestyle could modify this causal relationship between BMI and islet function.

Our results indicated that there was a causal relationship between BMI and insensibility in Chinese individuals. It’s an important role to demonstrating a causal relationship for scientists, but experiments could not be carried out because of some limitations. For example, some research variables, such as weight, sex, college, and BMI cannot be randomly assigned [17]. If we want to study the effect of those variables on health outcomes, we must eliminate co-occurrences to get closer to understanding the causal relationship. Individual are born with specific genetic variants that are randomly drawn from our parents according to Mendelian principles. This is a naturally occurring phenomenon that offers researchers a chance to randomly manipulate the variables that we cannot manipulate experimentally [18]. This is the basic idea of MR, and three key assumptions should be considered before using MR to study the data. Assumption 1: genes are randomly assigned among the population. In our study, 13 genetic loci were reported in a previous study, and we tested for Hardy–Weinberg equilibrium to determine randomness. All genetic loci were random in our samples. Assumption 2: some genetic loci influence the exposure factor [19]. In the present study, BMI is an exposure factor, and islet function is an outcome. Linear regression was performed to analyse the association between 13 genetic loci and BMI, and five SNPs (CDKAL1, MAP2K5, BDNF, FTO and SEC16B) that impacted BMI levels. Assumption 3: genes act on the outcome only by influencing the exposure. In Table 3, our results show that the GRS influenced insulin sensitivity, but after adjusting for BMI, the effect disappeared. This result indicates that the GRS influenced the IAI*10 via BMI rather than other channels.

BMI is an important index that reflects body obesity. Higher BMI levels (BMI ≥ 28 kg/m^2^) are a mark of obesity. An obese body has a mass of adipose tissue, which is a large adipose storage and dynamic endocrine organ in humans [20]. Adipose tissue releases free fatty acids, reactive oxygen species, and proinflammatory cytokines such as tumour necrosis factor α, interleukin 6, and interleukin 1 beta). These molecules increase in obesity-activated NF-κB and P38 MAPK signalling to target serine residues of the insulin receptor substrate protein, which are reduced in the liver and muscles. This may be the basic obesity-induced IR mechanism. In five SNPs influencing the GRS, all genes were associated with BMI in previous studies [21, 22]. In addition, some studies found that those SNPs were also directly related to IR. In the present study, we found that those genes were associated with IR via BMI, which may be a highlight of this study.

Daily diet and lifestyle have an essential role in health. Alcohol consumption has been associated with cardiovascular disease [23], effects on cognitive performance [24], and metabolism in numerous studies. Individuals who drank regularly had a higher IR level than individuals who abstained from alcohol, but Takemi et al. published the inverse conclusion in Japanese males [25]. In the present study, we found that drinkers had a lower insulin sensitivity than nondrinking individuals, and drinking status modified the association between BMI and IR. Specifically, the insulin sensitivity of drinkers was reduced with an increasing GRS, but the nondrinking group did not show this trend. There was an interaction effect for the IAI in groups with a different drinking status. Evidence shows that drinking alcohol impairs the liver of drinkers and produces steatohepatitis and fatty liver alcohol, which elevate hepatic IR and reduce hepatic insulin sensitivity [26]. In our questionnaire, we only investigated interviewees’ alcohol consumption in the last year. If an individual had abstained from alcohol for more than 1 year, we recorded this participant as nondrinking. Therefore, our results indicated a health benefit for those who abstained from alcohol for more than 1 year. Smoking is also a bad habit for people. Previous epidemiological studies have indicated that cigarette exposure is a risk factor for developing T2DM [27]. The underlying mechanisms may be that smoking impairs peripheral insulin sensitivity and pancreatic β-cell function [28, 29]. Our result, that smokers had a stronger negative relationship between BMI and insulin sensitivity, agrees with published studies. In the present study, we performed MR to analyse the effect of the interaction between BMI and lifestyle factors on islet function. Compared with conventional statistical methods, MR can substitute genetic loci for the exposure factor to analyse the interaction effect. Stable genetic loci can eliminate disturbance due to other factors. Hence, it is credible that the interaction effect result was from MR.

There are some limitations in this study. Dozens of BMI-associated genes have been reported by previous studies. However, our study only included 13 characteristic East Asian BMI-associated genes. Diet and lifestyle are important factors in IR, but this study only analysed alcohol consumption and smoking status in relation to IR. It should be noted that we found the interaction effect between GRS and drinking (smoking) on IAI, but because of lacking of quantity data of drinking (smoking), we could not perform dose–effect relationship to explore the interaction. According to our results, we reasonably believe that the individuals with higher quantity of smoking (drinking) have a worse IAI. In the further, we will increase the sample to explore the association between lifestyle and islet function.

## Conclusion

In summary, we found a causal relationship between BMI and IR, and that BMI was a significant risk factor for IR. This association was genetic, but lifestyle, such as drinking and smoking, could impair insulin sensitivity and increase IR.

## Data Availability

All data generated or analyzed during this study are included in this manuscript.

## Abbreviations

BMI: Body mass index
IR: Insulin resistance
T2DM: Type 2 diabetes mellitus
MR: Mendelian randomization
HOMA: Homeostasis model assessment
HOMAIR: Homeostasis model assessment insulin resistance
HOMA-β: Homeostasis model assessment -Beta cell function
IAI: Insulin sensitivity index
GRS: Genetic risk score
